# Health‐related quality of life in patients with Duchenne muscular dystrophy: a multinational, cross‐sectional study

**DOI:** 10.1111/dmcn.12938

**Published:** 2015-10-19

**Authors:** Erik Landfeldt, Peter Lindgren, Christopher F Bell, Michela Guglieri, Volker Straub, Hanns Lochmüller, Katharine Bushby

**Affiliations:** ^1^ Institute of Environmental MedicineKarolinska InstitutetStockholm; ^2^ Medical Management CentreDepartment of Learning, Informatics, Management and EthicsKarolinska InstitutetStockholmSweden; ^3^ GlaxoSmithKlineResearch Triangle ParkNCUSA; ^4^ Newcastle University John Walton Muscular Dystrophy Research CentreNewcastle upon Tyne; ^5^ MRC Centre for Neuromuscular DiseasesInstitute of Genetic MedicineNewcastle upon TyneUK

## Abstract

**Aim:**

To estimate health‐related quality of life (HRQOL) in patients with Duchenne muscular dystrophy (DMD).

**Method:**

HRQOL was assessed using the Health Utilities Index Questionnaire (HUI) and the Pediatric Quality of Life Inventory (PedsQL) neuromuscular module version 3.0 online. Results were stratified by disease stage (early/late ambulatory/non‐ambulatory) and caregivers’ perceptions of patients’ health and mental status.

**Results:**

A total of 770 patient–caregiver pairs (173 German, 122 Italian, 191 UK, and 284 USA) participated. Most caregivers (>84%) perceived their patients as happy/somewhat happy and in excellent/very good/good health, irrespective of current ambulatory class. In contrast, mean patient utility (reflecting public preferences: 0, dead; 1, perfect health) deteriorated with disease course, from 0.75 in early ambulatory males to 0.15 in the most severely affected patients. Mean patient PedsQL scores (0–100, higher score indicating better HRQOL) decreased from 80 to 57 across ambulatory classes.

**Interpretation:**

HRQOL in DMD, measured through public preferences, is substantially impaired in relation to the general population and significantly associated with disease progression. Still, most patients are perceived as happy and in good health by their caregivers, indicating that influential domains of HRQOL remain intact through the disease progression. Our findings emphasize the challenges in measuring HRQOL in a rare, progressive childhood condition such as DMD.

## What this paper adds

- Duchenne muscular dystrophy has a detrimental impact on health‐related quality of life (HRQOL) measured through public preferences.
- HRQOL measured through public preferences deteriorates with disease progression.
- Still, most caregivers perceive patients as happy and in good health.
- Our data suggest patients adapt to their changing health states.
- Measuring self‐perceived HRQOL in a rare, genetic disease is challenging.

This article is commented on by Pangalila on pages 435–436 of this issue.

**Abbreviations**
DMD: Duchenne muscular dystrophy
HRQOL: Health‐related quality of life
HUI: Health Utilities Index Questionnaire
PedsQL: Pediatric Quality of Life Inventory

The World Health Organization defines quality of life as individuals’ perceptions of their position in life in the context of the culture and value systems in which they live and in relation to their goals, expectations, standards, and concerns.^1^ Health‐related quality of life (HRQOL), in contrast, refers specifically to the individual's perception of the impact of health and illness on physical, mental, and social aspects of life.^2^, ^3^

Following improvement in survival in many chronic diseases, as well as increased requirements of evidence in terms of patient‐reported outcomes by regulatory agencies such as the US Food and Drug Administration, HRQOL has emerged as a central outcome both in clinical practice and in trials of new treatments. However, measuring HRQOL is challenging because aspects within physical, mental, and social life have different meanings and implications on HRQOL for each individual. In addition, children, adolescents, and adults have different reference systems and thus differ in their perceptions of HRQOL and its determinants.^2^ The individual's subjective perception of HRQOL may also change over time with age, as the disease progresses, or when new treatments become available.^3^, ^4^

A genetic, terminal illness in which survival has improved substantially during recent decades is Duchenne muscular dystrophy (DMD). DMD is a rare neuromuscular disease characterized by progressive muscle weakening, inevitable loss of independent ambulation, and a wide array of serious multisystem complications, including cardiomyopathy and respiratory muscle dysfunction.^5^ Untreated, mean age at death is about 19 years; however, after the implementation of respiratory support and proactive cardiac management, many patients with DMD now live to experience their third and sometimes fourth decade.^6^

Despite its central role in palliative DMD care, large series of HRQOL data in DMD are lacking. Existing evidence is usually restricted to one clinic or country and limited by small sample sizes (fewer than 60 cases) and/or inadequate stratification across age groups or stages of disease.^7^, ^8^, ^9^, ^10^, ^11^, ^12^, ^13^, ^14^, ^15^, ^16^, ^17^, ^18^ The objective of this study was to estimate HRQOL in patients with DMD at different stages of disease progression.

## Method

### Participants and outcome measures

This was a cross‐sectional, observational study of patients with DMD from Germany, Italy, the UK, and the USA identified through national DMD registries that form part of the global TREAT‐NMD Neuromuscular Network (http://www.treat-nmd.eu/). Patients were required to fulfil the following criteria: (1) male, (2) DMD diagnosis confirmed by genetic testing, and (3) age at least 5 years.

Eligible patients and one of their caregivers (e.g. a parent) were invited via e‐mail to complete the Health Utilities Index Questionnaire (HUI) and the Pediatric Quality of Life Inventory (PedsQL) neuromuscular module version 3.0 online.^11^, ^19^ The HUI is a generic HRQOL instrument encompassing 16 questions covering eight dimensions (hearing, speech, ambulation/mobility, pain, dexterity, self‐care, emotion, and cognition). Outcomes of the HUI are linked to preference measurements from the general public, representing mean community utilities ranging from 0 (dead) to 1 (perfect health). The HUI has been validated for proxy‐assessments in ages 5 years and older.^19^

The PedsQL is a construct encompassing disease‐specific HRQOL dimensions relevant to children with neuromuscular disorders, and has been validated for use with DMD.^11^ It consists of 25 questions covering three domains: (1) about my/my child's neuromuscular disease (17 questions related to the disease process and associated symptomatology); (2) communication (three questions related to the patient's ability to communicate with health care providers and others about his/her illness); and (3) about our family resources (five questions related to family financial and social support systems). For each question, respondents are asked to indicate to what degree a statement has been true during the previous month (five response categories, ranging from ‘never’ to ‘almost always’). Questions within the PedsQL are transformed to a scale ranging from 0 to 100, where a higher score indicates higher HRQOL. We asked patients to complete self‐report versions of the PedsQL (formats available for ages 5–7y, 8–12y, and 13–18y) and the caregivers to complete proxy‐report versions for the same age groups. We also asked the caregivers to indicate if their patients completed the PedsQL on their own (i.e. without any support, explanation, or suggestions); only those who did so were included for analysis.

Bi‐monthly reminders, as well as study invitations via regular post, were sent out by the participating TREAT‐NMD registries. All participants provided informed consent. Ethical approval for the study was granted from Ludwig‐Maximilians‐Universität München (Germany), Comitato Etico IRCCS E. Medea – Associazione La Nostra Famiglia (Italy), North East Research Ethics Service, NHS (UK), and the Western Institutional Review Board (USA). Approval was also obtained from the TREAT‐NMD Global Databases Oversight Committee.

### Statistical analysis

We assessed mean HUI‐derived utilities and mean PedsQL scores (caregiver proxy‐assessed and patient self‐assessed). We related our results to the progression of DMD by classifying patients into four groups defined in terms of current ambulatory status and age: (1) early ambulatory (approximate age 5–7y); (2) late ambulatory (approximate age 8–11y); (3) early non‐ambulatory (approximate age 12–15y); and (4) late non‐ambulatory (approximately 16y of age or older).^5^ We also stratified our estimates by the caregivers’ subjective ratings of their patients’ current health (five categories, ranging from ‘poor’ to ‘excellent’) and the caregivers’ subjective rating of their patients’ current mental status (five categories, ranging from ‘so unhappy that life is not worthwhile’ to ‘happy and interested in life’).

Agreement between patients’ self‐assessments and caregivers’ proxy‐assessments of PedsQL scores were investigated by estimating intraclass correlations from one‐way random‐effects models. We considered intraclass correlation coefficients less than 0.40 to indicate poor/fair agreement, 0.40 to 0.60 moderate agreement, 0.61 to 0.80 good agreement, and greater than 0.80 excellent agreement.^11^, ^12^

We assumed the sampling distributions of the sample means to be approximately normally distributed in accordance with the central limit theorem, and used Welch's analysis‐of‐variance models to compare estimates across strata and Welch's *t*‐tests for pairwise comparisons due to heterogeneous variances. We considered *p*<0.05 to be significant. All analyses were conducted in stata 12 (StataCorp LP, College Station, TX, USA).

## Results

A total of 770 patients with DMD completed the questionnaire together with one of their caregivers (Table 1). The overall study response rate was 42%. Caregivers had a mean age of 44 years (range 23–76) and 95% were parents to the participating patients. Additional details of the study sample have been published previously.^20^, ^21^

**Table 1:** Demographic statistics of the patients with Duchenne muscular dystrophy (*n*=770)

	*n* (proportion, %)
Country of residence (%)	
Germany	173 (22)
Italy	122 (16)
UK	191 (25)
USA	284 (37)
Mean age (SD), y	14 (7)
Ventilation support	126 (16)
Current ambulatory status (%)	
Early ambulatory (approximate age 5–7y)	155 (20)
Late ambulatory (approximate age 8–11y)	256 (33)
Early non‐ambulatory (approximate age 12–15y)	154 (20)
Late non‐ambulatory (approximate 16y of age or older)	205 (27)
Accommodation (%)	
Home of parents	726 (94)
Home of other relative	12 (2)
Own home	28 (4)
Group home or care facility	4 (1)
Current situation (%)	
Attending school, college, or university	591 (77)
Employed	21 (3)
Unemployed	158 (21)

Because of rounding, percentages might not add up to 100% exactly.

### Patient HRQOL assessed by the caregivers

**Figure 1:**
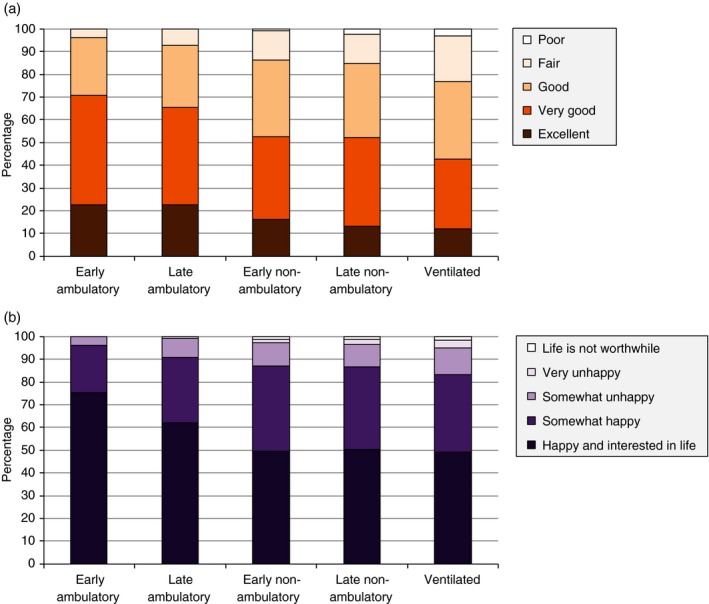
Caregivers' ratings of patients' current health (a) and mental status (b).

**Figure 2:**
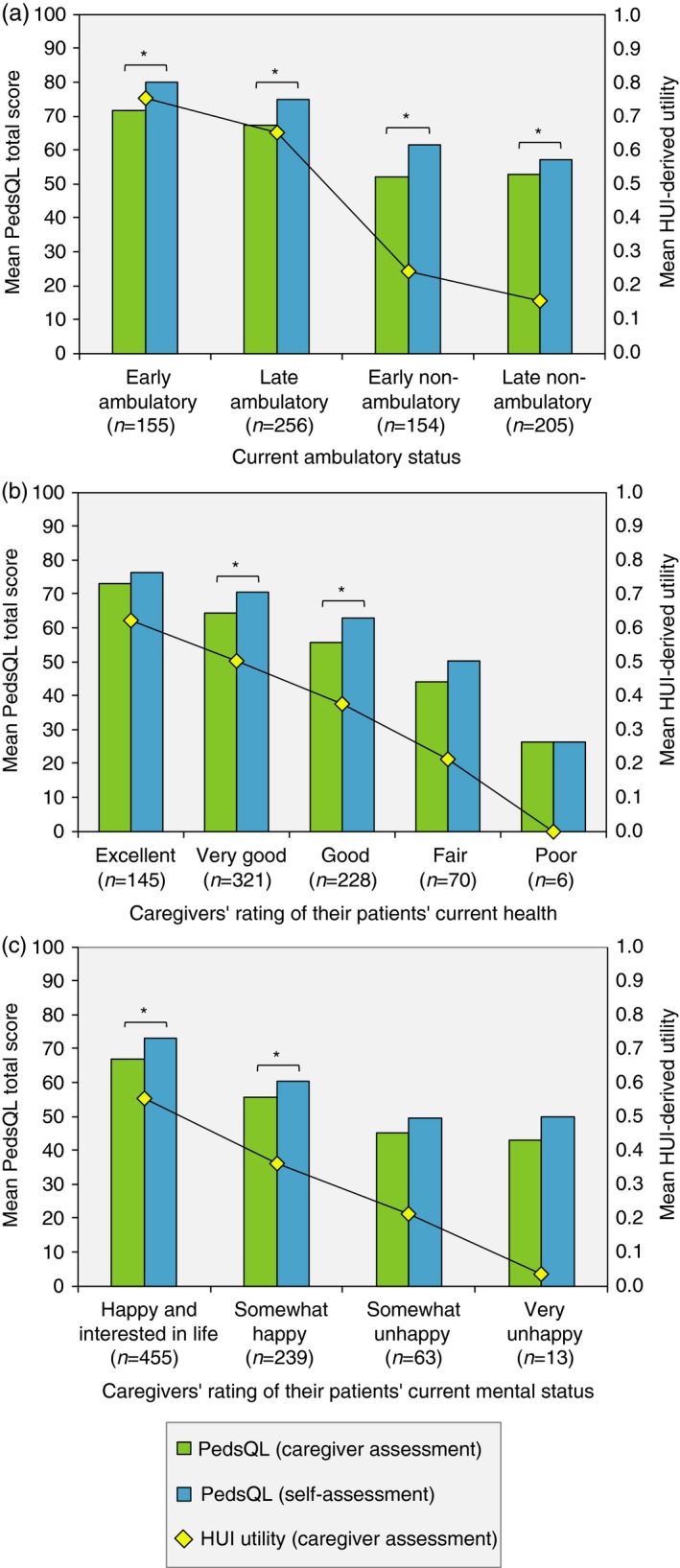
Self‐assessed and caregiver proxy‐assessed patient health‐related quality of life, by ambulatory status (a), patients' current health (b), and patients’ current mental status (c). *Statistically significant difference (at a 5% level) between patients’ self‐assessed Pediatric Quality of Life Inventory (PedsQL) total scores and caregivers’ proxy‐assessed total scores. The category ‘very unhappy’ also contains the category ‘so unhappy that life was not worthwhile’ (*n*=4 replies). Health Utilities Index Questionnaire (HUI).

Irrespective of current ambulatory class, most (>84%) patients were perceived as happy or somewhat happy, and in excellent, very good, or good health, by their caregivers (Fig. 1). In contrast, we observed a dramatic decrease in patient HRQOL, measured as HUI‐derived utilities representing public preferences, over the course of disease progression, ranging from 0.75 in early ambulatory patients (approximate age 5–7y) to 0.15 in late non‐ambulatory patients (approximately 16y of age or older) (*p*<0.001) (Fig. 2a). Mean HUI‐derived utility was also significantly associated with the caregivers’ rating of their patients’ current health (*p*<0.001) (Fig. 2b), and rating of their patients’ current mental status (*p*<0.001) (Fig. 2c). HUI‐estimates were comparable across countries (*p*=0.165).

Caregiver proxy‐assessed PedsQL total scores were significantly associated with ambulatory class (mean score range 52–72, *p*<0.001), but similar for early and late ambulatory patients, and early and late non‐ambulatory patients, and significantly associated with the caregivers’ rating of their patients’ current health (mean score range 26–73, *p*<0.001) and the caregivers’ rating of their patients’ current mental status (mean score range 43–67, *p*<0.001) (Fig. 2). The mean HUI‐derived utility and PedsQL total score in patients requiring ventilation support was estimated at 0.10 and 48 respectively. Across countries, proxy‐assessed mean PedsQL total scores ranged between 58 and 65 (*p*=0.001).

### Patient self‐assessment of their HRQOL

A total of 518 (67%) patients (mean age 15y, range 5–43y) were able to complete the PedsQL without help from their caregivers. The mean self‐assessed PedsQL total score was estimated to be between 57 and 80 across ambulatory classes (*p*<0.001) (Fig. 2a), between 26 and 76 across the caregivers’ rating of their patients’ current health (*p*<0.001) (Fig. 2b), and between 50 and 73 across the caregivers’ rating of their patients’ current mental status (*p*<0.001) (Fig. 2c). The mean score in patients requiring ventilation support was 52.

Although the mean PedsQL score was similar across countries (*p*=0.105), there were differences in the areas reported to be of most concern. German and Italian patients indicated most frequent problems with turning at night, waking up tired, difficulties in using the bathroom, and planning family activities like vacations. In the UK sample, in addition to tiredness, patients indicated most frequent problems in their families not getting enough rest and planning family activities. US patients indicated most problems with pain in their legs, tiredness, and explaining their illness to other people. Approximately 20% of German patients indicated that they often or always lacked the equipment they needed. The corresponding proportions of the Italian, UK, and US sample were 6%, 17%, and 11% respectively.

### Comparison of self‐ and proxy‐assessment of patient HRQOL

Patients’ self‐assessed PedsQL total scores were consistently higher than caregivers’ proxy‐assessed scores across the investigated strata (Fig. 2). Intraclass correlation coefficients between the patients’ and caregivers’ total scores were estimated between 0.56 and 0.79 across ambulatory classes (indicating moderate to good agreement), 0.64 and 0.94 across caregivers’ rating of their patients’ current health (good to excellent agreement), and 0.64 and 0.77 across caregivers’ rating of their patients’ current health (good agreement). For the pooled sample, the intraclass correlation coefficient was estimated as 0.78 (good agreement) (Table 2).

**Table 2:** Comparison of self‐ and proxy‐assessments of patient health‐related quality of life

	Patient self‐assessment score	Caregiver proxy‐assessment score	Intraclass correlation coefficient
PedsQL total score	67 (65–68)	61 (60–63)	0.78 (0.74–0.81)
About my/my child's neuromuscular disease	66 (64–68)	64 (62–65)	0.84 (0.81–0.87)
Communication	68 (65–70)	60 (57–62)	0.67 (0.62–0.72)
About our family resources	67 (64–69)	54 (52–56)	0.57 (0.50–0.63)

Data presented as mean (95% CI). PedsQL, Paediatric Quality of Life Inventory neuromuscular module version 3.0.

## Discussion

Children with DMD are born into a life of chronic illness, inexorably increasing physical disability and dependence, and knowledge of an inevitably premature death. Compared with the reference points of a healthy peer, this life would appear bleak. However, from the perspective of the affected individuals, who do not know any other life, they may perceive their well‐being differently. With these premises in mind, we set out to measure HRQOL in children, adolescents, and young adults with DMD. In addition to informal assessment of current health and mental status, we used two validated instruments: one generic linked to public preferences and one disease‐specific, in search of a more complete picture of HRQOL along the progression of DMD.

Despite the considerable morbidity associated with the disease, the vast majority of patients with DMD in our sample were perceived as happy and in good health by their caregivers, with only minor variation across ambulatory classes or needs of ventilation support for survival. Contrary to these findings, our estimates of mean proxy‐assessed patient HUI‐derived utility (representing HRQOL as measured by members of the public) was strongly negatively associated with disease progression and substantially lower than reference values for Canadian children and young adults (0.94).^22^ For non‐ambulatory patients (approximate age ≥12y), our mean utility estimate (0.19) was also lower than published estimates for other chronic, non‐progressive conditions such as cerebral palsy (0.28), Down syndrome (0.36), deafness (0.42), and autism spectrum disorder (0.43), as well as previous utility estimates for muscular dystrophy and spinal muscular atrophy (0.39).^23^ The minimum meaningful change in HUI scores has been estimated to be between 0.03 and 0.05.^19^ The mean utility change from the early ambulatory to the late non‐ambulatory class of 0.60 observed in our study thus indicates a loss in HRQOL of extraordinary magnitude.

There are several possible reasons for the observed discrepancy between HUI‐derived utilities and the caregivers’ ratings of patients’ health and mental status across ambulatory classes (e.g. low utility estimates for patients perceived to be happy and in good health). First, given the inherently progressive nature of DMD, patients and their caregivers may be subject to a phenomenon known as ‘response shift’ or ‘well‐being paradox’, which is the process of adapting to a changed health state and of accommodating illness.^2^ Caregivers may also adjust their perception of their patients’ HRQOL based on disease history as well as the anticipated disease trajectory (where the current health state may appear relatively good given the expected well‐being in more advanced stages of DMD). Lastly, since DMD is a genetic condition, patients are not familiar with a life free from disease, and do not have the same references as healthy individuals against which to compare their current situation. Findings similar to ours have been reported for individuals with, for example, cerebral palsy, who have been shown to have comparable HRQOL as their healthy peers,^24^ but also very poor HRQOL as measured through public preferences using the HUI.^23^

Previously published data on the association between DMD progression and HRQOL are limited by small sample sizes and based on many different ratings scales, which make comparisons between studies challenging. Bray et al. show in two studies of 34 and 35 Australian patients with DMD respectively that patient HRQOL is significantly negatively associated with physical functioning but uncorrelated with psychosocial well‐being.^7^, ^15^ Comparable findings were reported by Kohler et al.^16^ in a study of 35 Swiss patients, Uzark et al.^12^ in a study of 117 US patients, and Elsenbruch et al.^17^ in a study of 50 German patients. In the last two studies, older males/adolescents were found to have even better psychosocial health than their younger counterparts. A negative association between HRQOL and wheelchair use and ventilation support was also found in a study of 27 Italian patients with DMD by Baiardini et al.^8^ Our results expand on these previous reports and provide valuation of patient HRQOL from the perspective of the public appropriate for use in health economic evaluations as recommended by the National Institute for Health and Care Excellence in the UK. Our findings should also be helpful in informing decisions about clinical trial endpoints defined in terms of HRQOL outcomes given the observed pattern of deterioration across the disease course; however, they emphasize that any measurement of self‐perceived HRQOL in a paediatric disease such as DMD needs to be interpreted with caution.

We estimated the mean patient self‐assessed PedsQL total score to be between 26 and 80 across the investigated strata. Only two studies have, to our knowledge, assessed HRQOL in patients with DMD using the neuromuscular version of PedsQL, and they estimated the mean parent‐proxy score at 60 and 53 respectively.^11^, ^13^ However, interpreting and comparing PedsQL outcomes is challenging because the scoring algorithm does not take into account that the impact on HRQOL from the questions and response categories included in the instrument is likely to vary across respondents. For example, a child indicating that he always has problems explaining his disease to other people is attributed the same loss in HRQOL as a child indicating the he always feels tired, although the impact on HRQOL may be very different. In addition, the impact on HRQOL of, for example, always feeling tired would be expected to vary across individuals. In other words, the PedsQL measures HRQOL on an ordinal scale and fails to incorporate the heterogeneity in the determinants of HRQOL across respondents, as well as the potential interaction between different questions and response categories and HRQOL.

In our sample, one‐third of all patients (252/770) were unable to complete the PedsQL without help from their caregivers, and for this subgroup the mean PedsQL total score was slightly higher but not significantly different than scores for those who completed the instrument on their own (69 compared with 67, *p*=0.079). The caregiver proxy‐assessed mean PedsQL total score for these patients was also higher (63 compared with 61, *p*=0.089), as well as mean HUI‐derived patient utility (0.52 compared with 0.42, *p*<0.001). Data on why some patients could not provide self‐assessments were not collected as part of the study, but possible reasons include cognitive problems, which are prevalent in DMD, as well as issues in interpreting language and meaning of questions among younger respondents (even though we used age‐specific formats), as noted in studies of other paediatric populations.^2^

Although our findings generally were comparable across countries, we found some differences in PedsQL results. A potential reason for this finding includes differences in general population HRQOL (e.g. generally lower HRQOL for those living in the UK compared with the USA) as suggested by, for example, EuroQol EQ‐5D value sets,^25^ but the differences cannot be fully analysed in our data set and warrant further investigation. Other factors of importance may relate to cultural differences in care experiences and expectations. Moreover, in the PedsQL, about one in every five German patients indicated that they often or always lacked the equipment they needed. This finding is in agreement with our previous report of discrepancies between real‐world care and DMD care guidelines, where, for example, only 29% of German patients reported having access to any orthosis compared with 65%, 65%, and 70% in Italy, the UK, and the USA respectively.^21^ Affordable access to medical devices and aids is central to help maintain HRQOL and allow patients with DMD to participate in society as their physical functioning deteriorates.

Historically, in research of HRQOL in paediatric populations, proxy‐reports by, for example, caregivers or physicians have been used when the child is too ill or deemed cognitively incapable of making reliable assessments. Today, with the development of age‐specific instruments, the consensus is that children can and should report their own HRQOL. However, the use of proxy‐reports is also encouraged as they may provide additional, complementary HRQOL data.^2^ In our study, patients consistently ranked their HRQOL more favourably than their caregivers. Still, we noted good to excellent agreement between proxy‐ and self‐assessments of patient HRQOL, with the exception of the PedsQL cluster ‘About our family resources’ (where agreement was moderate). Thus, our findings indicate less discrepancy than previous research reporting poor to fair,^11^, ^12^ poor to fair to moderate,^7^ moderate,^13^ and moderate to good agreement.^14^ Potential reasons include differences between studies in, for example, the distribution of patients across disease stages, patient–caregiver relationships (parents vs more distant relatives), and extent of caregiver involvement in the daily life of the patient with DMD.

A limitation of our study relates to the generalizability of results. Patients were recruited via TREAT‐NMD registries with a mean response rate of 42%, and as participation in the registries is voluntary and family‐initiated, we cannot rule out a degree of selection bias. However, given that the collected clinical and epidemiological data were characteristic for the different patient groups (not reported), that our sample was evenly distributed across the defined ambulatory classes, and that the distribution of ages was similar for responders and non‐responders, the discrepancy between the sample and study populations should be limited. It is also worth pointing out that the response rate among those who actually received a study invitation would be expected to be higher as a result of, for example, lost invitations due to recent changes to e‐mail addresses and spam filters.

In summary, we show that HRQOL in DMD, measured through public preferences, is substantially impaired in relation to general population reference values, strongly negatively associated with disease progression, and in good agreement with the caregivers’ subjective perceptions of their patients’ current health and mental status. Still, most children and young adults with DMD are rated as happy and in good health by their caregivers, irrespective of current ambulatory class, indicating that some domains of HRQOL remain intact through the progression of DMD. Our findings also emphasize the challenges in measuring HRQOL in paediatric populations in general, and a rare, progressive childhood disease such as DMD in particular.
